# Machine Learning for Intensive Care Unit Length-of-Stay Prediction: A Simulation-Based Approach to Bed Capacity Management

**DOI:** 10.1177/0272989X251406639

**Published:** 2025-12-26

**Authors:** Sara Garber, Yarema Okhrin

**Affiliations:** Department of Statistics and Data Science, Faculty of Business and Economics, University of Augsburg, Augsburg, Germany; Centre for Interdisciplinary Health Research, University of Augsburg, Augsburg, Germany; Department of Statistics and Data Science, Faculty of Business and Economics, University of Augsburg, Augsburg, Germany; Department of Economics and Statistics, Linnaeus University, Växjö, Sweden

**Keywords:** intensive care management, capacity control, explainable artificial intelligence, medical decision support, predictive analytics, Monte Carlo simulation

## Abstract

**Background:**

While machine learning (ML) models are increasingly used to predict outcomes in health care, their practical effect on health care operations, such as bed capacity management, remains underexplored. There is a variety of traditionally used evaluation metrics to analyze ML models; however, decision makers in health care settings require a deeper understanding of their implications for resource management. Traditional performance measures often fail to provide this practical insight.

**Methods:**

In this work, we conduct a simulation study to evaluate the impact of ML-driven length-of-stay (LOS) predictions on intensive care unit (ICU) bed capacity management. Two classification models differing in terms of explainability and interpretability, logistic regression (LR) and extreme gradient boosting (XGB), are applied to predict ICU-LOS. We use the HiRID dataset containing high-frequency data of more than 33,000 patients. The predictions of the ML models are integrated into a simulation framework that replicates real-world ICU bed management, allowing for the assessment of the practical implications of using these algorithms in a clinical setting.

**Results:**

The application of both classification models results in improved capacity control regarding the key performance indicators in the simulation study, with XGB outperforming LR. While LR leads to slight overoccupancy in the ICU, slight underoccupancy can be observed when XGB is applied.

**Conclusion:**

Our study bridges the gap between predictive accuracy and practical application by emphasizing the importance of evaluating ML models within the context of ICU capacity management. The simulation-based approach offers a more relevant assessment for health care practitioners, providing actionable insights that go beyond classical performance measures and directly address the needs of decision makers in clinical practice.

**Highlights:**

- We apply multiple classification models for ICU-LOS prediction using time-series data. This approach enables an update of the initial prediction resulting in the possibility of efficiently managing intensive care capacities.
- We present a simulation-based approach to evaluate ML algorithms and their impact on bed capacity management in real-world clinical settings.
- Our work provides in-depth insights into the impact of using ML techniques as decision support systems in the ICU and can lead to increased acceptance in practice.

The German health care system is not crisis resistant and not sufficiently prepared for the consequences of climate change or pandemics: this is the conclusion of a report by the German Council of Experts on the Assessment of Developments in the Healthcare Sector, which cited the inadequate use of digital options as one of the key weaknesses.^1^ The vulnerability of the system, especially in the event of crisis-related challenges, has been highlighted in recent years by the COVID-19 pandemic, which has led to an increased burden on the entire German health care system, particularly hospitals. In February 2021, 10% of COVID-19 patients in Germany were hospitalized, of which 33% were estimated to be treated in the intensive care unit (ICU).^2^ The enormous increase in the number of patients requiring care presented a major challenge to physicians and highlighted the relevance of forecasting and managing the need for intensive care capacity. In this context, the length of stay (LOS) in the ICU (ICU-LOS), describing the treatment period of a patient, is crucial. A precise prediction can contribute to optimizing the care of critically ill patients, the efficient use of resources, and the improvement of patient care. Furthermore, knowledge of the individual LOS enables enhanced management of intensive care capacities as the treatment duration of a patient can be considered, for example, in high-load situations when transferring patients to other hospitals.

In this context, the application of artificial intelligence and in particular machine learning (ML) methods as a subarea can contribute to an accurate prediction. In the literature, both regression and classification algorithms are used to predict ICU-LOS. When using regression methods, the ICU-LOS is regarded as a continuous variable, and the aim is to make as accurate a prediction as possible for each patient.^3–8^ When applying classification algorithms, binary classification is often applied to differentiate between “regular” and “prolonged” ICU-LOS.^3,5,9–18^ However, the definition of prolonged ICU-LOS varies depending on the study, as datasets with different patient characteristics and different use cases are considered. The threshold value is often set at 7 d,^9,12,17^ although distinctions such as between ≤2 d and >2 d^15^ or ≤10 d and >10 d^5^ are also defined in the literature. In practice, multiclass classification is often useful for making more concrete predictions of the ICU-LOS for each patient and to be able to plan capacities accordingly. In the existing literature on multiclass classification, differences can also be observed with regard to the number of classes selected as well as their size. Iwase et al.^19^ distinguished between 3 classes, short (<1 wk), medium (1–2 wk), and long (>2 wk), whereas Chen et al.^20^ selected 4 classes, 4 d, 4 to 7 d, 7 to 10 d, and >10 d, and Alabbad et al.^21^ differentiated between 9 classes, whereby intervals of 5 d were chosen. The studies on multiclass classification also highlight distinct variations in sample sizes, ranging from 353 (Chen et al.^20^) to 895 (Alabbad et al.^21^) to 12,747 (Iwase et al.^19^) patients. Overall, there is a strong heterogeneity in the applications of ML models for ICU-LOS prediction in the literature. This reflects the fundamentally different approaches that, although potentially reasonable depending on the context, hinder comparability and may not be suitable in all settings. Moreover, no forecasts specifically supporting a daily planning level for ICUs including resource management perspectives are provided. A subdivision into classes of several days can enable a better performance of the forecast; however, a more accurate prediction of the ICU-LOS, for example, on a daily planning level, can be desirable for the hospital from a management perspective.^22–24^

In addition, the focus of the existing literature is primarily on the performance of the algorithms measured by state-of-the-art performance indicators. Of particular interest to decision makers in practice is the real-world effect of applying these models. For instance, in the case of ICU-LOS prediction, the insights gained are crucial for optimizing intensive care capacity and effectively managing bed occupancy. By accurately predicting patient stay durations, health care providers can enhance the allocation of resources, streamline patient flow, and ultimately improve overall care delivery in the ICU and the entire hospital. Although state-of-the-art performance indicators enable a comparison of individual algorithms, they provide only a limited indication of the effects of an actual application in the ICU. This aspect is often neglected in the existing literature. However, the literature, particularly in the fields of operations research and management science, frequently features studies that bridge predictive analytics with practical operations. In the context of ICU management, several papers have explored the application of simulation methods^25–29^ as well as the integration of ML and artificial intelligence with simulation approaches^30–33^ in intensive care settings. A comprehensive overview of how operations research and management science can inform ICU capacity planning is provided in the review by Bai et al.^34^ In general, further analysis with respect to the evaluation of an application of the algorithms in practice is essential due to the importance of the combination of artificial intelligence driven by ML and medical decision making.^35^

In our work, we focus on this essential interface and apply 2 classification algorithms for ICU-LOS prediction differing in terms of transparency and explainability, namely, logistic regression (LR) and extreme gradient boosting (XGB), on a real-world intensive care dataset. The ICU-LOS was divided into 4 classes, that is, 1 d, 2 d, 3 d, and ≥4 d, allowing us to focus on day-based capacity planning and operational needs in a clinical setting. At each point in time (i.e., days of treatment), a prediction is made as to whether a patient will continue to be treated in the ICU for 1 more day or longer. Therefore, the ICU-LOS is not only predicted shortly after the beginning of treatment in the ICU but also updated daily. In addition to state-of-the-art performance indicators and decision curve analysis, we use a Monte Carlo simulation for further analysis and evaluation of the classification algorithms. In the simulation study, we model ICU bed capacity management based on the predictions generated by the classification algorithms. This approach offers deeper and more practical insights than traditional key performance indicators do, providing physicians with actionable information that directly addresses the challenges of resource allocation and patient care. Our study offers in-depth insights into the real-world effects of implementing classification algorithms on a daily planning level in the ICU, which is crucial for effective capacity management and informed medical decision making in hospitals.

## Methods

### Dataset

Our study was based on the high time-resolution ICU dataset (HiRID, version 1.1.1) available on PhysioNet,^36,37^ including more than 33,000 patients admitted to the ICU in the Bern University Hospital in Switzerland from January 2008 to June 2016. Descriptive statistics and further information about the dataset as well as the preprocessing procedure applied can be found in the original publication by Hyland et al.^38^ We used the preprocessed dataset containing 18 meta-variables consisting of observational and pharmaceutical data with a 5-min time grid. In addition, 3 general variables (i.e., age, admission time, and sex) were included, resulting in 21 variables considered for predicting ICU-LOS. The target variable was rounded up to whole days, as from a practical perspective it is useful to forecast on a daily basis to enable appropriate planning and management of capacities in the hospital. The patients were divided into 4 classes, resulting in the following number of patients per class: 18,629 patients with an ICU-LOS of 1 d, 6,722 patients with an ICU-LOS of 2 d, 2,623 patients with an ICU-LOS of 3 d, and 5,931 patients with an ICU-LOS of 4 d or longer. With regard to the simulation study, a treatment period of 4 d was assumed for all patients in the last class.

### Classification Algorithms

Two classification algorithms, XGB and LR, were used to predict each patient’s ICU-LOS. To ensure comparability, in addition to the 2 models (XGB and LR[c]), we also included a simplified LR model (LR[s]) based on a selection of basic clinical covariates as well as a naïve forecast. This allows for comparison with clinical practice, since ICU managers typically rely on basic observable patient characteristics for planning while also providing the standard benchmark with a naïve forecast that is commonly used in the literature. In general, the transparency and explainability of the algorithms play a central role in the application of ML models, particularly in medicine. In this context, a distinction can be made between black-box models such as XGB and white-box models such as LR. Black-box models are mathematically complex, which makes it difficult for users to understand them in practice.^39^ Due to their high complexity, these models usually provide precise predictions, but the internal decision-making rules and mechanisms are not transparent. In contrast, white-box models are easier to understand by experts in the field of application.^39^ However, the predictions are often less precise due to the lower complexity of the models. In general, the consideration of transparency and explainability is particularly relevant in the context of the application of ML algorithms in health care, as neglecting this aspect in clinical decision support systems poses a threat to fundamental ethical values in medicine.^40^

Our approach consists of a combination of 3 binary classification algorithms to predict ICU-LOS. This proceeding allowed the time-series data to be adequately considered, as updated patient information can be used for further prognosis. Shortly after a patient was admitted to the ICU, an initial prediction was made as to whether this patient will need to be treated for 1 d or longer than 1 d ( LOS=1∨LOS>1 ). The information collected in the first 40 min after admission to the ICU was used as input for the initial prognosis of a patient. This time period was chosen because all patients in the dataset were still being treated in the ICU during this period, and therefore, all data were available. Depending on the feature, the mean value, maximum value, or a binary variable indicating whether a certain treatment took place is used for each meta-variable. The prediction at the initial point in time of treatment, ${\hat{p}}_{i,0},$ was made for all patients $i\in I_{0}$ receiving treatment. A prediction was made for each point in time τ∈T={0,1,2} ; therefore, a distinction was also made for the consecutive points in time, which leads to the following generically formulated classification:

(12) $${\hat{p}}_{i,\tau }=\left\{ \begin{array}{cc} 0\; & \mathrm{for}\;\mathrm{LOS}=\tau +1\\ 1\; & \mathrm{for}\;\mathrm{LOS}\;>\;\tau +1 \end{array}\right.\;\forall \tau \in T,i\in I_{\tau }$$

**Figure 1:**
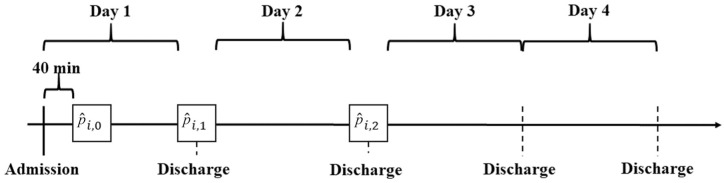
Graphical representation of the classification procedure ( ${\hat{p}}_{i,0}$ – prediction in τ = 0, ${\hat{p}}_{i,1}$ – prediction in τ = 1, ${\hat{p}}_{i,2}$ – prediction in τ = 2).

For the predictions at the 2 consecutive points in time, that is, ${\hat{p}}_{i,1}$ ( LOS=2∨LOS>2 ) and ${\hat{p}}_{i,2}$ ( LOS=3∨LOS>3 ), an additional binary classification was performed for the patients still receiving treatment in the ICU, that is, $i\in I_{1}$ and $i\in I_{2}$ , respectively. This ensured that predictions were generated only for patients who were still undergoing treatment and for whom the necessary data for further forecasting were fully available. For further classification, the information previously collected for the patients was used and thus included the data from 24 and 48 h (informative time horizon), respectively, in the ICU. A graphical representation of our procedure outlining the informative time horizon, predictive time horizon, and prediction time (represented by τ∈T) is presented in Figure 1.

Due to the 5-min time grid, the data available for each patient are very extensive. Therefore, a linear regression model was fitted for each of the existing meta-variables, and the respective parameters (i.e., intercept and slope) were used as the input for the classification algorithm. For each prediction, that is, ${\hat{p}}_{i,0}$ , ${\hat{p}}_{i,1}$ , and ${\hat{p}}_{i,2}$ , both XGB and the LR models were applied. Thus, a total of 9 classification models were created.

For each model, a 5-fold cross-validation procedure was used in the training process. Usually, this procedure is applied for hyperparameter tuning to select the best model. In our study, we modified the classic procedure with the aim of obtaining a prognosis for each patient in the dataset and thus being able to use the entire dataset in the simulation study. The dataset was randomly divided once in 5 different folds, each consisting of 20% of the patients treated in the ICU. For each submodel, 4 folds (i.e., 80% of the patients) were used as training dataset, and the remaining fold (i.e., 20% of the patients) was used as test dataset. Consequently, 5 submodels, each using a different fold as test dataset, were created for each of the 9 classification models to be able to use an out-of-sample prediction for each individual patient in the subsequent simulation study. Logistic Lasso regression was used for the LR method owing to its advantages in feature selection and model complexity, which have made it a common choice in the statistical literature. For XGB, the hyperparameters were set to basic values traditionally regarded as reasonable to ensure fair comparability across the individual submodels. Detailed specifications of the parameters are provided in the accompanying R code for this article. Our approach enables the possibility of carrying out the extensive simulation study based on all data available, which is particularly relevant for an application in practice, as most of the datasets accessible in the health care sector are comparatively small, and it is crucial to adequately use all the information provided.

### State-of-the-Art Performance Evaluation

The classification models were evaluated using state-of-the-art performance indicators, that is, area under the receiver-operating characteristic curve (AUROC), sensitivity, specificity, precision, scaled Brier score, F1 score, and accuracy. These performance measures are used as standard when comparing ML models and are intended to enable good comparability. In our study, this applies both when analyzing the selected ML algorithms and when comparing the performance quality of the individual submodels at the points in time considered. The use of classic evaluation methods enables good comparability with other studies, even if the data basis used can have an enormous influence on the performance. Furthermore, variable importance plots as well as calibration plots are used to assess model performance for the classification algorithms.

In addition, we applied decision curve analysis (DCA) to assess the practical utility of the algorithms. This approach accounts for the clinical impact of decision-making outcomes.^41^ The foundation of these considerations is the idea of calculating the potential cost and benefit of a clinical intervention.^42^ In our context, this clinical intervention was defined as intensive care treatment extending for more than 1 additional day. The choice of the decision threshold, a probability value ranging from 0 to 1, is of central importance,^43^ as the necessity of an intervention is predicted in the model from this point onward. The theoretical relationship between benefit and selected thresholds can be leveraged, enabling model evaluation against the commonly used clinical benchmarks “treat all” and “treat none” without requiring additional data.^41^ It is important to note that DCA is not intended to determine the optimal threshold.^44^ Instead, it provides a method to evaluate whether the model offers clinical benefit, while the appropriate range of thresholds should always be defined according to practical requirements.^44^ In our example, we assumed that a threshold of 0.5 is generally appropriate for management purposes. However, hospital-specific circumstances must be considered; for example, in high-demand situations, lower thresholds may be more practical to facilitate timely management decisions, such as transferring patients to other facilities. In addition, thresholds greater than 0.5 may be relevant in this context and are therefore also examined further. From a practical perspective, the choice of the appropriate threshold depends on whether the risk of an empty bed or an overbooked patient is considered to be higher. Overall, an evaluation of the threshold range from 0.25 to 0.75 appeared practical for a real-world application of the classification models for predicting ICU-LOS.

### Simulation Study

In addition to the evaluation by means of the classically used performance measures, a comprehensive simulation study was subsequently carried out to analyze the algorithms in greater depth. We applied a Monte Carlo simulation, as this method can provide valuable insights into the effects of data-based decision support in the ICU.^45^ We simulated ICU capacity management by incorporating predictions generated by the classification models, specifically focusing on how these predictions influence hospital bed management. By using ICU-LOS predictions derived from the algorithms, this method enables a thorough and comprehensive evaluation of their impact on ICU bed utilization and highlights the practical implications for resource allocation within the hospital setting.

At the beginning of the simulation study, the input parameters, that is, number of runs ( R=1,000 ), number of consecutive points in time considered ( T=10 ), and number of beds (B=50) , were defined. The Department of Intensive Care Medicine of the Bern University Hospital from which the dataset used originates is an interdisciplinary 60-bed unit^36^; however, the desired capacity utilization was set at 50 in our simulation study, as it cannot be assumed in practice that all available capacities will be scheduled without a buffer. After initializing the input parameters, an initial bed occupancy at the initial point in time t=0 was generated for each simulation run r and each ICU-LOS prediction model m∈M (i.e., XGB, LR, simplified LR, and a naïve classifier), assigning the patient to the majority class in every binary prediction based on the proportions in the dataset, by randomly selecting 50 patients from the dataset. Subsequently, the currently used ICU-LOS prediction and thus the current point in time of treatment τ was assigned to each patient i∈I based on a discrete uniform distribution; for example, for a patient with an actual ICU-LOS of 2 d, there is an equal probability of being assigned to either τ=0 or τ=1 . Only the predictions actually made were used; for example, if a patient i is receiving treatment for 1 d in the ICU and then discharged, the prediction at the point in time of treatment τ=0 (i.e., ${\hat{p}}_{i,0})$ , was used. This procedure was chosen because in practice it will be in only exceptional cases that the ICU is empty, and 50 patients are admitted and starting their intensive care treatment at the same time.

**Figure 2:**
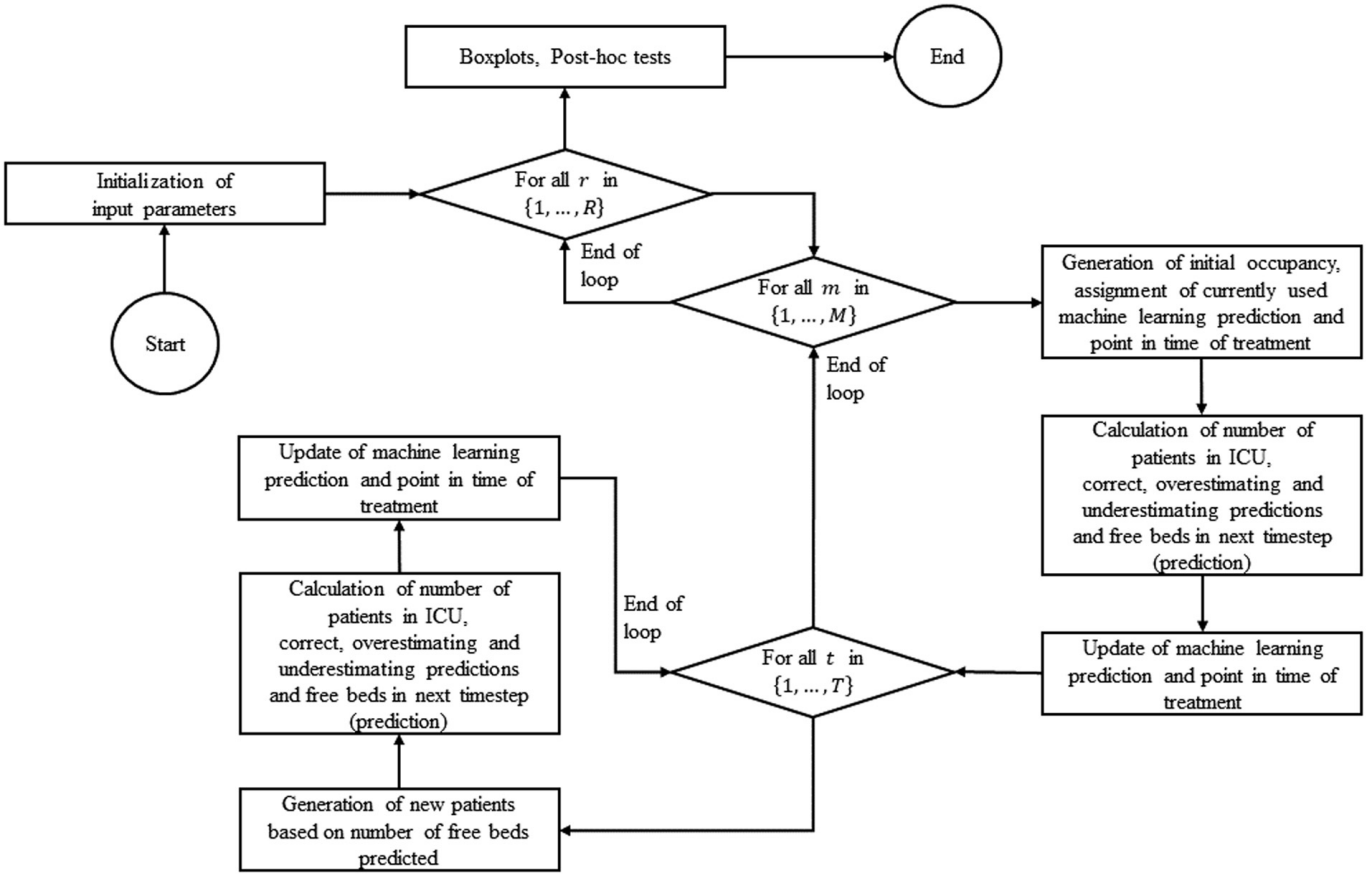
Flowchart of the Monte Carlo simulation setup in R.

Then, the number of patients being treated in the ICU was calculated, which is always 50 at the time of initial occupancy t=0 , as well as the number of correct, overestimating, and underestimating predictions. This approach was chosen to evaluate not only the pure number of patients treated but also how many patients treated at each specific point in time received an accurate, overestimated, or underestimated prediction of ICU-LOS when the respective classification algorithm was applied. This served as an additional evaluation and validation of the results. In the next step, the number of beds becoming available at the next point in time was determined based on the currently used ICU-LOS prediction for each patient receiving treatment in the ICU. The prediction used and the time of treatment for each patient were now updated (i.e., the passing of a day was simulated). New patients were now generated at all consecutive points in time. Therefore, the predicted number of free beds in the ICU was used according to the forecast. However, the current number of patients was considered; that is, if, for example, not all beds were occupied, more patients would be admitted at the next point in time by this number of free beds in addition to the admissions due to predicted discharges and vice versa. This approach ensures a realistic representation of human–machine interaction. Following the generation of the newly admitted patients, the number of patients currently receiving intensive care treatment; the number of correct, overestimating, and underestimating predictions; as well as the number of beds available at the next point in time were calculated again. The prediction used and the time of treatment as well as the current point in time were updated. The procedure described was repeated for each subsequent point in time considered. Afterward, the number of patients and number of correct, overestimating, and underestimating predictions in each run and each point in time were used for further analysis. For this purpose, the mean values were calculated, and boxplots were created for a visualized representation of the results. In addition, an analysis of variance and post hoc tests were carried out to additionally validate the results derived from the simulation study. All implementation was performed using the programming language R (version 4.3.1) and its development environment RStudio (version 2023.9.0.463) on a Windows system. The flowchart for a detailed representation of the simulation setup in R is shown in Figure 2.

## Results

### State-of-the-Art Performance Evaluation

An initial evaluation of the ML models is carried out using the classic performance measures AUROC, sensitivity, specificity, precision, scaled Brier score, F1 score, and accuracy. For all binary classification models, the longer ICU-LOS is defined as the positive class (class 1). The AUROC, which we use as the primary performance measure in our state-of-the-art evaluation, is highest for the XGB model for all predictions. In addition, the AUROC is higher for the LR(c) model than for the LR(s) model. When using the LR(s) model, the AUROC is 0.63, 0.67, and 0.63 for the predictions ${\hat{p}}_{i,0}$ , ${\hat{p}}_{i,1}$ , and *${\hat{p}}_{i,2}$ *, respectively. In contrast, the values are 0.67, 0.69, and 0.66 for the LR(c) and 0.78, 0.74, and 0.68 for the XGB model. There are also substantial differences with regard to the other performance measures. The sensitivity varies considerably within the different models with values of 0.33, 0.71, and 0.99 for the LR(s) model; 0.43, 0.70, and 0.96 for the LR(c) model; and 0.66, 0.73, and 0.91 when using the XGB model for the predictions ${\hat{p}}_{i,0}$ , ${\hat{p}}_{i,1}$ , and *${\hat{p}}_{i,2}$ *. Fluctuations can also be observed when looking at the specificity, which is 0.84, 0.54, and 0.02 as well as 0.83, 0.58 and 0.08 when using the LR models, respectively, but 0.76, 0.62 and 0.20 when using the XGB model. Overall, when examining sensitivity and specificity, it is evident that sensitivity increases while specificity decreases over time for all classification models. The accuracy, which is likely not clinically relevant but included due to its frequent use in the literature, is statistically significantly higher than the no-information rate for XGB and both LR models, at points in time *τ=0* and *τ=1 *. In contrast, this is not the case at point in time τ=2 , for which there is no statistically significant difference in comparison to the no-information rate at a level of significance of 0.05. Those results are derived from a 1-sided binomial test, which is standard practice in R for evaluating the performance of the prediction models. However, it should be noted that due to the comparatively large sample size, clinically meaningless differences might appear statistically significant. All performance measures, including 0.95 confidence intervals and the no-information rate, are shown in Table 1 for the classification algorithms, LR(s), LR(c), and XGB, as well as the predictions at the individual points in time for each patient receiving intensive care treatment, ${\hat{p}}_{i,0}$ , ${\hat{p}}_{i,1}$ , and ${\hat{p}}_{i,2}$ . The AUROC is shown for each submodel (i.e., performance is measured for each individual fold representing 20% of the data available) and for the entire dataset. This tabular presentation enables a comparison of the performance of the individual submodels created as well as the individual points in time but also in relation to all patients contained in the dataset for a comprehensive evaluation of the performance of the classification algorithms, LR(s), LR(c), and XGB, for ICU-LOS classification.

**Table 1:** Area under the Receiver-Operating Characteristic Curve (AUROC), Sensitivity (SE), Specificity (SP), Precision (PRE), Scaled Brier Score (BS), F1 Score (F1), and Accuracy (ACC) in Comparison with the No-Information Rate (NIR) (1-Tailed Binomial Test) per the Machine Learning Model and Point in Time of Prediction

	LR(s)			LR(c)			XGB		
	${\hat{p}}_{i,0}$	${\hat{p}}_{i,1}$	${\hat{p}}_{i,2}$	${\hat{p}}_{i,0}$	${\hat{p}}_{i,1}$	${\hat{p}}_{i,2}$	${\hat{p}}_{i,0}$	${\hat{p}}_{i,1}$	${\hat{p}}_{i,2}$
AUROC	0.63 [0.62, 0.63]	0.67 [0.66, 0.68]	0.63 [0.63, 0.64]	0.67 [0.67, 0.68]	0.69 [0.68, 0.70]	0.66 [0.65, 0.67]	0.78 [0.78, 0.79]	0.74 [0.73, 0.74]	0.68 [0.67, 0.69]
$\mathrm{AURO}C_{1}$	0.62	0.64	0.64	0.68	0.67	0.67	0.78	0.72	0.69
$\mathrm{AURO}C_{2}$	0.62	0.66	0.64	0.67	0.69	0.67	0.79	0.74	0.67
$\mathrm{AURO}C_{3}$	0.64	0.67	0.62	0.67	0.70	0.64	0.77	0.74	0.67
$\mathrm{AURO}C_{4}$	0.63	0.68	0.62	0.67	0.70	0.66	0.79	0.74	0.67
$\mathrm{AURO}C_{5}$	0.62	0.67	0.64	0.67	0.70	0.67	0.78	0.74	0.69
SE	0.33 [0.32, 0.24]	0.71 [0.70, 0.72]	0.99 [0.98, 0.99]	0.43 [0.42, 0.43]	0.70 [0.69, 0.71]	0.96 [0.96, 0.97]	0.66 [0.65, 0.67]	0.73 [0.72, 0.74]	0.91 [0.90, 0.92]
SP	0.84 [0.84, 0.85]	0.54 [0.53, 0.55]	0.02 [0.02, 0.03]	0.83 [0.82, 0.83]	0.58 [0.56, 0.59]	0.08 [0.07, 0.09]	0.76 [0.75, 0.76]	0.62 [0.60, 0.63]	0.20 [0.19, 0.22]
PRE	0.63 [0.62, 0.64]	0.66 [0.65, 0.67]	0.70 [0.69, 0.71]	0.67 [0.66, 0.68]	0.68 [0.67, 0.69]	0.70 [0.69, 0.71]	0.69 [0.68, 0.70]	0.71 [0.70, 0.72]	0.72 [0.71, 0.73]
BS	0.04	0.08	0.04	0.08	0.11	0.06	0.24	0.17	0.08
F1	0.43	0.68	0.82	0.52	0.69	0.81	0.68	0.72	0.81
ACC	0.61* [0.61, 0.62]	0.63* [0.62, 0.64]	0.69 [0.68, 0.70]	0.65* [0.64, 0.65]	0.65* [0.64, 0.65]	0.69 [0.68, 0.70]	0.71* [0.71, 0.72]	0.68* [0.67, 0.68]	0.70 [0.69, 0.70]
NIR	0.55	0.56	0.69	0.55	0.56	0.69	0.55	0.56	0.69

${\hat{p}}_{i,0}$ , initial prediction; ${\hat{p}}_{i,1}$ , prediction after 1 d of treatment; ${\hat{p}}_{i,2}$ , prediction after 2 d of treatment.

* Statistically significant values to a significance level of 0.05.

**Figure 3:**
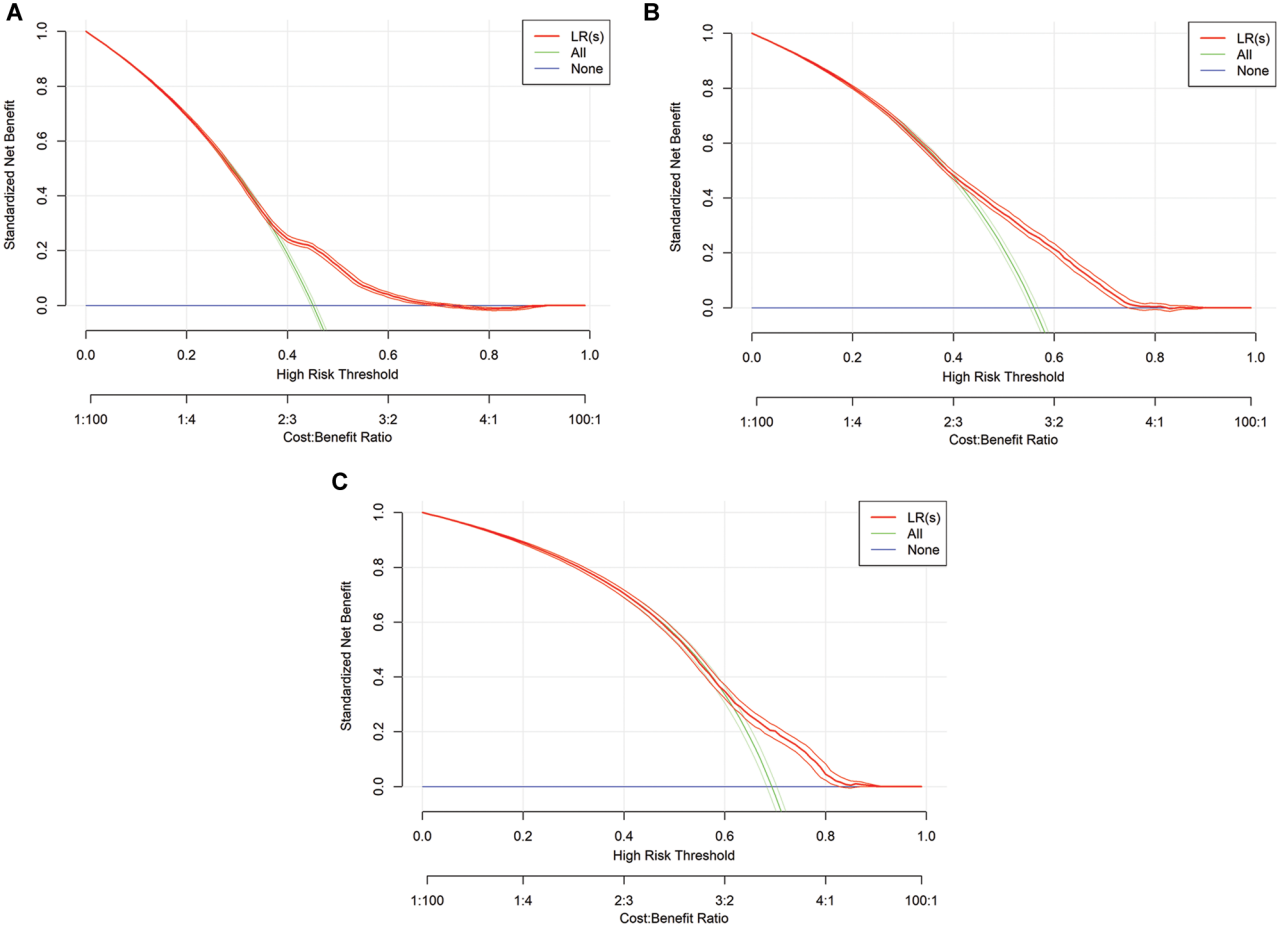
Graphical representation of decision curve analysis for logistic regression (LR) models. s, simple: τ = 0, τ = 1, τ = 2.

**Figure 4.**
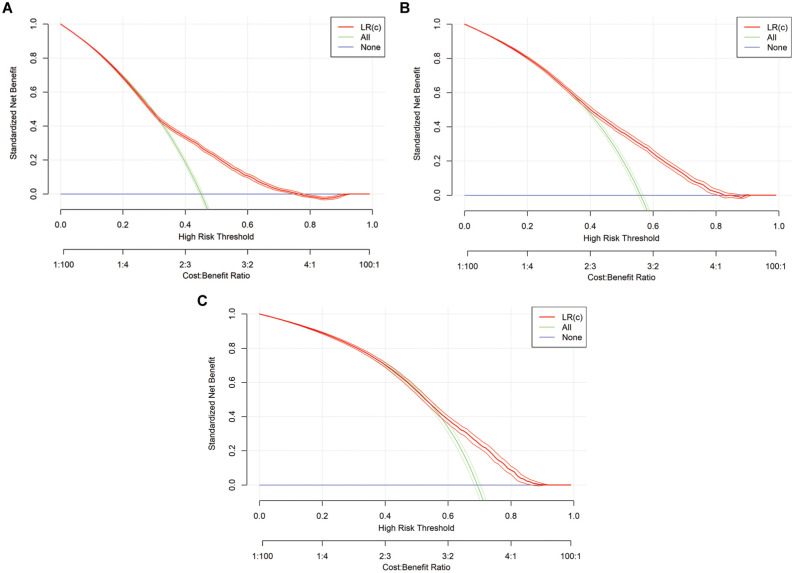
Graphical representation of decision curve analysis for logistic regression (LR) models. c, complex: τ = 0, τ = 1, τ = 2.

**Figure 5:**
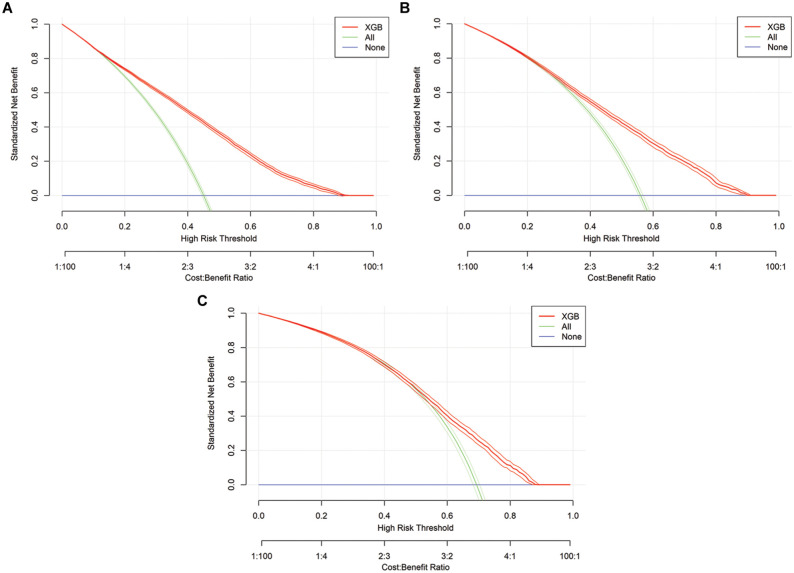
Graphical representation of decision curve analysis for extreme gradient boosting (XGB) models in all time steps: τ = 0, τ = 1, τ = 2.

In addition to the presented performance measures, DCA was conducted to assess the benefits of the models, with a particular focus on the predefined threshold range of 0.25 to 0.75. Overall, the benefit demonstrates notable differences when using the classification models compared with the “treat all” and “treat none” strategies. At points in time τ=0 and *τ=1 *, the standardized net benefit is higher when using LR(c) and XGB, respectively, as compared with the 2 benchmark strategies, provided a threshold of 0.5 is applied. The DCA is presented visually in Figure 3 for LR(s), Figure 4 for LR(c), and Figure 5 for XGB.

**Figure 6:**
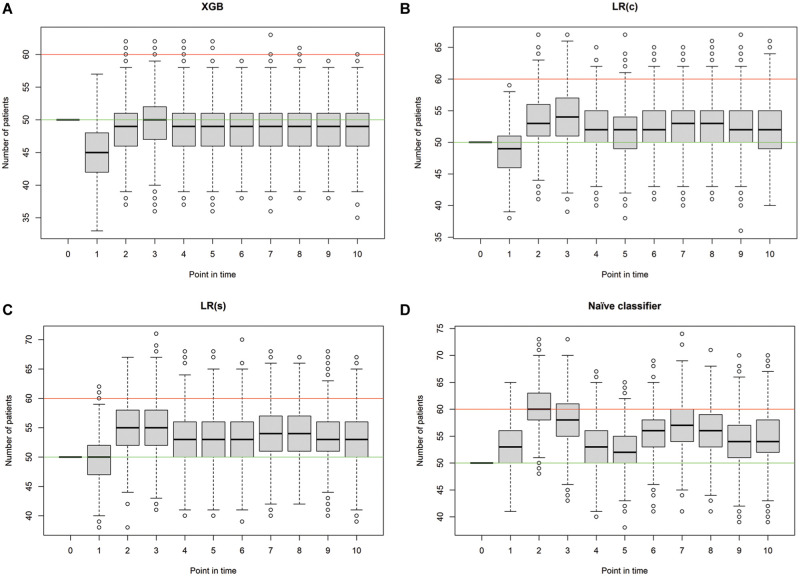
Simulated occupancy of the intensive care unit per classifier and point in time. Green line, number of beds used for initial occupancy (50); red line, maximal number of beds available (60). XGB, extreme gradient boosting: τ = 0, τ = 1, τ = 2, τ = 3.

In addition, we used variable importance plots (Supplementary Figures 7–15) as well as calibration plots (Supplementary Figures 16–18) to assess the model performance for all time steps, which can be found in the Appendix.

### Simulation Study

In the simulation study, the number of patients receiving intensive care treatment as well as the number of correct, overestimating, and underestimating predictions are used as key performance indicators to compare the 4 prediction models (i.e., naïve classifier, simple LR, complex LR, and XGB). At the initial point in time, t=0 , all 50 beds that were defined as input parameter in the Monte Carlo simulation are fully occupied. In contrast, the consecutive points in time show differences in the 4 classifiers with regard to the mean value of the number of patients treated. For example, at the consecutive point in time t=6 , the average number of patients treated is 55.55 when using the naïve classifier. However, this number is lower when applying ML models to predict ICU-LOS. On average, 53.42 patients and 52.21 patients are treated when using the LR(s) and LR(c) model, respectively, and 48.62 when using the XGB model. For the more complex LR(c) and XGB models, the mean number of patients treated is closer to 50 for most points in time than when using the LR(s) model and the naïve classifier. In the case of LR(c), the number is mostly higher than the specified number of beds and slightly lower in the case of XGB. In total, the number of patients receiving intensive care treatment is closest to 50 when applying the XGB model. Furthermore, there are differences between the algorithms with regard to the number of correct, overestimating, and underestimating predictions. The mean over all simulation runs r∈R of all key performance indicators used to compare the 4 classifiers throughout the period considered is depicted in Table 2.

**Table 2:** Mean Number of Patients in the ICU ( $N_{\mathrm{pat}}$ ) and Mean Number of Correct ( $N_{\mathrm{cor}}$ ), Overestimating ( $N_{\mathrm{ov}}$ ), and Underestimating ( $N_{\mathrm{un}}$ ) Predictions per Point in Time in the ICU ( t∈T ) per Classifier and Naïve Prediction (NP) over All Simulation Runs r∈R

	t = 0	t = 1	t = 2	t = 3	t = 4	t = 5	t = 6	t = 7	t = 8	t = 9	t = 10
NP											
$N_{\mathrm{pat}}$	50.00	52.82	60.39	57.91	52.80	52.46	55.55	56.79	55.70	54.31	54.53
$N_{\mathrm{cor}}$	34.45	30.01	35.26	34.92	33.44	32.86	33.74	34.40	34.06	33.52	33.36
$N_{\mathrm{ov}}$	6.36	6.21	8.61	10.10	8.45	7.02	7.51	8.35	8.67	8.13	7.88
$N_{\mathrm{un}}$	9.19	16.60	16.52	12.90	10.91	12.57	14.30	14.04	12.97	12.66	13.29
$LR_{s}$											
$N_{\mathrm{pat}}$	50.00	49.79	54.81	54.93	53.14	52.51	53.42	53.98	53.76	53.40	53.34
$N_{\mathrm{cor}}$	34.68	31.63	35.03	35.69	35.69	34.86	35.30	35.62	35.70	35.35	35.29
$N_{\mathrm{ov}}$	7.76	6.64	7.40	8.00	7.40	7.04	7.00	7.23	7.26	7.29	7.25
$N_{\mathrm{un}}$	7.56	11.53	12.39	11.24	10.06	10.61	11.13	11.14	10.80	10.76	10.80
$LR_{c}$											
$N_{\mathrm{pat}}$	50.00	48.65	53.30	53.65	52.41	51.81	52.21	52.62	52.58	52.50	52.11
$N_{\mathrm{cor}}$	35.11	32.56	35.57	35.80	35.84	35.59	35.51	35.70	35.85	35.90	35.42
$N_{\mathrm{ov}}$	8.12	6.29	6.92	7.60	7.18	6.78	6.81	7.02	6.91	7.03	7.06
$N_{\mathrm{un}}$	6.77	9.80	10.81	10.25	9.40	9.44	9.89	9.90	9.82	9.57	9.63
XGB											
$N_{\mathrm{pat}}$	50.00	45.02	48.63	49.44	48.91	48.58	48.62	48.72	48.71	48.64	48.74
$N_{\mathrm{cor}}$	35.72	32.01	34.63	35.17	35.25	35.24	35.24	35.17	35.17	35.00	35.15
$N_{\mathrm{ov}}$	9.63	6.94	7.03	7.41	7.08	6.95	6.95	7.06	7.08	7.07	6.97
$N_{\mathrm{un}}$	4.65	6.07	6.97	6.85	6.58	6.39	6.42	6.49	6.46	6.57	6.62

Therefore, the ICU occupancy was measured for each simulation run to provide a detailed overview in addition to the mean values per point in time. Furthermore, we used post hoc tests to test the statistically significant differences in the number of patients receiving treatment in the ICU. In the initial time step, there is no statistically significant difference since the number of initial patients is 50 for each model. In the consecutive time steps, there is a statistically significant difference in most cases, except in t=4 for LR(c) and NP, LR(s) and NP, as well as LR(s) and NP for t=5 . In addition, boxplots are used for the visual representation of the results. Across all classifiers, the maximum capacity of 60 patients is exceeded, although this occurs more frequently with the naïve classifier compared with the ML models. Using the XGB algorithm, only outliers exceed this value. The boxplots representing the number of patients receiving intensive care treatment per point in time for each classifier are depicted in Figure 6. The green line represents the number of beds initialized in the simulation study, which is equal to 50, and the red line is the maximal number of beds available in the ICU, which amounts to 60.

## Discussion

The use of state-of-the-art performance measures shows that the XGB classifier outperforms the LR classifiers with regard to the AUROC for the predictions ${\hat{p}}_{i,0}$ , ${\hat{p}}_{i,1}$ , and ${\hat{p}}_{i,2}$ . At the first prediction shortly after the admission to the ICU, ${\hat{p}}_{i,0}$ , the sensitivity of the LR(c) model is considerably lower, leading to an underestimation of the ICU-LOS. In the simulation study, this leads to an overestimation of the number of beds becoming available at the next point in time and therefore to the admission of too many patients. This is also reflected when considering the key performance indicator $N_{\mathrm{un}}$ as the number of underestimating predictions is higher when using the LR(c) classifier. At point in time τ=1 , the sensitivity of both classification models is comparable. The specificity is lower when applying the XGB algorithm for ${\hat{p}}_{i,0}$ but again similar for ${\hat{p}}_{i,1}$ . The difference in ${\hat{p}}_{i,0}$ leads to an increased overestimation of the ICU-LOS when using the XGB model, which is also reflected in the simulation study and its key performance indicators. An overestimation of the ICU-LOS leads to an underestimation of the number of beds becoming available and thus to a lower number of patients being admitted at the next point in time. At point in time τ=2 , there is no statistically significant difference between the accuracy of both classification models and the no-information rate. Therefore, it is difficult to differentiate between an ICU-LOS of 3 d and 4 d. The application of DCA demonstrates that at a threshold of 0.5, both ML models provide a clinical benefit at the time points t=0 and t=1 compared with the benchmark strategies “treat all” and “treat none.” Furthermore, it is evident that the XGB model delivers clinical benefits across a broader range of thresholds, for prediction ${\hat{p}}_{i,0}$ particularly within the previously defined relevant range from 0.25 to 0.75. This enables greater flexibility in application overall. In general, a threshold of 0.5 appears reasonable, since both unoccupied beds and overestimated patient admissions entail drawbacks from a resource management perspective. An unoccupied bed represents an opportunity cost, as another patient who could have been treated is turned away despite available capacity. Conversely, admitting more patients than capacity allows can also lead to negative consequences, such as treatment delays for the patient in question or others. For elective patients scheduled for ICU after major planned surgeries, rescheduling can be necessary if capacity constraints arise. While such rescheduling may be feasible, it entails significant short-term organizational effort. In general, if the patient ICU-LOS tends to be underestimated, this may lead to bottlenecks, potentially resulting in the need to transfer patients to other hospitals. Conversely, overestimating ICU-LOS leads to wasted resources, a particularly severe issue in times of staffing shortages and high-workload situations. Therefore, selecting an appropriate threshold should be context dependent, taking into account the specific ICU and the mix of elective and emergency patients it handles. Overall, both under- and overutilization of capacity should be avoided in practice, first and foremost to minimize the negative consequences for patients and to ensure the best possible care. In addition, from an operational perspective, the efficient use of limited resources is desirable, as it benefits medical staff and health care providers while also helping to reduce costs. More details on potential outcomes are to be found in the literature on cost-effectiveness analyses in ICU settings.^46–49^

The simulation study shows that the application of both classification models is advantageous compared with the naïve prediction as well as the LR(s) model. Overall, the number of patients treated in the ICU using the XGB classifier is closest to the specified number of beds to be occupied by means of capacity management. However, the application of the LR(c) classifier also shows promising results, especially in comparison with the naïve classifier. When planning the ICU capacity using the predictions of the LR model, the average number of patients treated is usually slightly greater than 50; when using the XGB model, it is always less than 50. The use of the classification models also demonstrates benefits in terms of the maximum number of patients that can be accommodated in the ICU. This is especially evident with the XGB classifier, as only outliers have values exceeding 60. If the number of patients exceeds the number of free beds available and thus not all patients can no longer receive intensive care treatment, a transfer to other hospitals or, in extreme cases, triage must be carried out.^45^ It is therefore particularly relevant in practice to plan sufficient buffers to ensure the ability to treat emergency patients and provide medical care for all patients. As explained in detail in the previous part of the discussion, there is always a tradeoff to consider between free or unused capacity, which generates costs for the hospital, and ensuring patient care, which is central to the health care system. Both in the use of state-of-the-art performance measures and in the simulation study, it is evident that XGB outperforms LR. However, the advantage of white-box models such as LR lies in the fact that decision makers in the health care system can more easily understand how the predictions are made, which can lead to higher acceptance in practice. The use of a simulation study can also contribute to higher acceptance, as it clearly shows the effects of the algorithms in practical application. This is essential for ML models to be actually applied in practice and thus enhance patient care. In summary, both classification algorithms, LR(c) and XGB, perform well and can therefore contribute to improved decision support in the ICU.

Overall, it is important to emphasize that incorporating a simulation study within the context of using ML predictions in hospitals can provide a comprehensive evaluation of their impact on capacity management. This approach enables a nuanced analysis of how these forecasts influence resource allocation and operational efficiency, thereby offering valuable support to decision makers in optimizing hospital operations. In future research, this approach could be further refined and expanded. One potential development is the integration of simulation within the training process of the algorithms, allowing for real-time evaluation based on capacity control metrics. By focusing on the practical impact of these algorithms on resource management, rather than relying solely on traditional evaluation measures, this method could offer a more tailored and effective adaptation for real-world applications. This shift would ensure that ML models are not only accurate but also optimized for the specific operational needs of health care environments. Another approach in this context could be to focus more on the role of explainability of the algorithms. This might involve considering doctors’ perspectives when using such forecasts and the benefits of visualizing parameters in white-box models. For black-box models, incorporating methods that enhance the transparency of complex algorithms can be useful. This is particularly relevant for establishing data-driven decision support systems in practice. Ensuring that the needs and preferences of the decision makers in practice are included is crucial for successful implementation. Furthermore, this work focuses on short-term planning since we use time-series data that allow for daily updates of the prediction. An alternative approach for medium- and long-term forecasting would be to generate a day-based prediction at the outset without subsequent updates. This may be appropriate, for instance, when time-series data are not available or when incorporating up-to-date patient information is not technically feasible. In general, the application of prediction models for medium- and long-term planning in ICUs could be further investigated in future research.

This study is subject to some limitations. First, the ICU-LOS of the patients is divided into 4 different classes, but in practice, the target variable is continuous and not discrete. This dichotomization of the continuous outcome inevitably entails a loss of information. However, from an operational management perspective, planning in discrete time units is practical and meaningful. Capacity planning in hospitals is often done on a daily basis, for which our ML models can provide valuable support. In addition, our approach enables a good comparison of the algorithms within the framework of the simulation study since our dataset contains time-series data allowing for dynamic updates throughout the treatment process. Second, we assume a maximum ICU-LOS of 4 d for the simulation study, which can be exceeded in practice. Third, we used a minimal tuning approach for hyperparameters in the XGB model to ensure comparability across the submodels. However, extensive hyperparameter tuning could improve the performance. Fourth, the characteristics of the patients can differ depending on the ICU considered. For example, differences can result from the proportion of elective and emergency patients as well as the size and medical specialties in the hospital. Therefore, a direct transfer of the results to other ICUs is possible to only a limited extent. However, our work provides a detailed explanation of the advantages of using classification algorithms and their simulation-based analysis for medical decision making in hospitals. Fifth, this study is based on retrospective data, but to actually implement the algorithms in practice, the data must be continuously collected and stored, which requires sufficient technical conditions and adequate software in the hospital.

## Conclusion

Our study provides a simulation-based analysis of 2 algorithms differing in terms of transparency and explainability, LR and XGB, for ICU-LOS classification. The predictions are made both shortly after the beginning of treatment and as a daily update. The results show that the application of a Monte Carlo simulation can provide valuable insights into the effects of the classification algorithms in case of application in practice. In further research, the consideration of data-driven mathematical statistical analyses from an interdisciplinary perspective is essential to enable the most efficient management of available resources in the ICU. This approach has the potential to help ensure the adequate preparation for future challenges for the health care system such as pandemics or the consequences of climate change.

## Supplemental Material

sj-docx-1-mdm-10.1177_0272989X251406639 – Supplemental material for Machine Learning for Intensive Care Unit Length-of-Stay Prediction: A Simulation-Based Approach to Bed Capacity Management Supplemental material, sj-docx-1-mdm-10.1177_0272989X251406639 for Machine Learning for Intensive Care Unit Length-of-Stay Prediction: A Simulation-Based Approach to Bed Capacity Management by Sara Garber and Yarema Okhrin in Medical Decision Making

sj-docx-2-mdm-10.1177_0272989X251406639 – Supplemental material for Machine Learning for Intensive Care Unit Length-of-Stay Prediction: A Simulation-Based Approach to Bed Capacity Management Supplemental material, sj-docx-2-mdm-10.1177_0272989X251406639 for Machine Learning for Intensive Care Unit Length-of-Stay Prediction: A Simulation-Based Approach to Bed Capacity Management by Sara Garber and Yarema Okhrin in Medical Decision Making
