# Supplementary material for: Machine Learning for Intensive Care Unit Length-of-Stay Prediction: A Simulation-Based Approach to Bed Capacity Management
Source: Med Decis Making. 2025 Dec 26;46(3):355–70. doi: 10.1177/0272989X251406639 (PMC12976099; doi:10.1177/0272989X251406639)
Supplement: sj-docx-1-mdm-10.1177_0272989X251406639 – Supplemental material for Machine Learning for Intensive Care Unit Length-of-Stay Prediction: A Simulation-Based Approach to Bed Capacity Management [file sj-docx-1-mdm-10.1177_0272989X251406639.docx]

**Strengthening the Reporting of Empirical Simulation Studies (STRESS)**

**System Dynamics guidelines STRESS-SD**

| **Section/Subsection** | **Item** | **Recommendation** | |
| --- | --- | --- | --- |
| 1. **Objectives** |  |  | |
| Purpose of the model | 1.1 | Explain the background and rationale for the model.  Introduction; Section Methods, Subsections Classification Algorithms and Simulation Study | |
| Model Outputs | 1.2 | Describe all outcome variables that are reported. Include details on how they are calculated during the model run. These might be as simple as detailing which specific stocks/levels are of key interest or may involve detailing equations.  Section Methods, Subsection Simulation Study | |
| Experimentation Aims | 1.3 | If the model has been used for policy analysis (user-defined experiments) and policy formulation (multiple experiments to obtain best policy), state the research questions that it was used to answer.   1. Policy based analysis – Provide a name and description of each policy tested, providing a rationale for the choice of policies and parameters employed. Ensure that item 2.3 (below) is completed. 2. Design of experiments – Provide details of the design and the parameters that will be used. This for example, may be to perform a sensitivity analysis. 3. Simulation Optimisation - Provide full details of what is to be optimised and the parameters that will be included and the algorithm that will be used. Where possible provide a citation of the algorithm/calibration method.   Section Methods, Subsection Simulation Study; only partially applicable | |
| 1. **Logic** |  |  | |
| Base model overview diagram | 2.1 | Provide one or more causal loop, stock and flow (a.k.a level and rate) or equivalent diagrams to describe the basic logic of base model to readers. Avoid complicated diagrams in the main text. To aid readers understanding authors should document the key feedback loops that drive system behaviour.  Section Methods, Subsection Simulation Study | |
| Base model logic | 2.2 | Give details of the base model logic in terms of feedback loops. This could be text to explain the overview diagram along with extra details including any use of system archetypes.  Section Methods, Subsection Simulation Study | |
| Scenario logic | 2.3 | Give details of the logical difference between the base case model and policies, scenarios and experiments. This could be incorporated as text or where differences are substantial could be incorporated in the same manner as 2.1.  Section Methods, Subsection Simulation Study | |
| Algorithms | 2.4 | Provide detail on any algorithms, functions or equations that mimic complex or manual processes in the real world. E.g. scheduling of arrivals/appointments/operations/maintenance. Sufficient detail should be included (or referred to in other published work) for the algorithms to be reproducible. For clarity, it is recommended that algorithms are represented as pseudo-code.  Section Methods, Subsection Simulation Study; the use of pseudo-code is deemed unnecessary since the R-code used in this study will be made available via GitHub | |
| Components | 2.5 | 2.5.1 Stocks/Levels | Give details of all stocks within the simulation including a description of their role in the model. Provide the stocks units and make sure that all inflows and outflows can easily be identified (note that in a large model a diagram is unlikely to sufficiently clear for other researchers to use. Please consider tables in addition to any diagrams).  Section Methods, Subsection Simulation Study |
|  |  | 2.5.2 Flows/Rates | List all flows within the model along with units and equations. Describe the role of flows in the model e.g. if they act a delay.  Section Methods, Subsection Simulation Study |
|  |  | 2.5.3 Constants / Converters / auxiliary variables | List all variables within the model and detail their equations (if applicable) including units.  Section Methods, Subsection Simulation Study |
|  |  | 2.5.4 Graphical functions/lookup tables | List and detail all graphical functions within the model and describe their data sources.  Section Methods, Subsection Simulation Study; only partially applicable |
|  |  | 2.5.5 Sources and Sinks | Give details of the model boundaries i.e. all infinite sources and sinks within the model.  Section Methods, Subsection Simulation Study; only partially applicable |
| 1. **Data** |  |  | |
| Data sources | 3.1 | List and detail of all data sources. Sources may include:   - Interviews with stakeholders, - samples of routinely collected data, - prospectively collected samples for the purpose of the simulation study , - Public domain data published in either academic or organisational literature. For published literature include the reference.   All data source descriptions should include details of the sample size, date ranges and use within the study.  Section Methods, Subsection Dataset | |
| Pre-processing | 3.2 | Provide details of any data manipulation that has taken place before its use in the simulation, e.g. interpolation to account for missing data or the removal of outliers.  Section Methods, Subsections Dataset and Classification Algorithms | |
| Input parameters | 3.3 | List of all input variables in the model, provide a description of its use and include parameter values. For stochastic inputs provide details of any continuous, discrete or empirical distributions used along with all associated parameters. Give details of all time dependent parameters, correlation and any graphical functions.    Clearly state:   - Base case data - Data use in experimentation (where different from the base case). - Where optimisation or design of experiments has been used, state the range of values that parameters can take.   Where theoretical distributions are used, state how these were these selected and prioritised above other candidate distributions.  Section Methods, Subsection Simulation study | |
| Assumptions | 3.4 | Where data or knowledge of the real system is unavailable what assumptions are included in the model? This might include parameter values, distributions or flow logic within the model.  Section Methods, all Subsections | |
| 1. **Experimentation** |  |  | |
| Initialisation | 4.1 | List all initial values of stocks and auxiliary variables within the model.  Provide details of empirical or theoretical distributions used, if initial values are varied over multiple runs of the model (e.g. for calibration or optimisation).  Section Methods, Subsection Simulation study | |
| Run length | 4.2 | Detail the run length of the simulation model and time units  Section Methods, Subsection Simulation study | |
| Estimation approach | 4.3 | If the SD model includes stochastic inputs report if multiple replications or an alternative approach has been used in estimating model outputs.  For model calibration or optimisation report the algorithm/search method used and the number replications (runs) of the simulation model. Section Methods, Subsection Simulation study | |
| 1. **Implementation** |  |  | |
| Software or programming language | 5.1 | State the operating system and version and build number.  State the name, version and build number of commercial or open source SD software that the model is implemented in (e.g. Vensim, iThink, InsightMaker). Many modern SD packages provide specialised stocks to act as delays e.g. conveyers, queues and ovens. Provide details all specialised stocks and where they are used in the model.  State the name and version of general-purpose programming languages used (e.g. Python 3.5.2). Where packages, frameworks and libraries have been used provide all detailed including version numbers.  Section Methods, Subsection Simulation Study & R-Code | |
| Random sampling | 5.2 | State the algorithm used to generate random samples with in the software/programming language used e.g. Mersenne Twister.  Section Methods, Subsection Simulation Study & R-Code | |
| Model execution | 5.3 | Report the integration method used along with time step settings.  Section Methods, Subsection Simulation study | |
| System Specification | 5.4 | State the model run time and specification of hardware used. This is particularly important for large scale models that require substantial computing power.  Only partially applicable, see Section Methods | |
| 1. **Code Access** |  |  | |
| Computer Model Sharing Statement | 6.1 | Describe how someone could obtain the model described in the paper, the simulation software and any other associated software (or hardware) needed to reproduce the results. Provide, where possible, the link and DOIs to these.  Section Methods, Subsection Dataset/Data availability statement | |
