# Supplementary material for: Machine Learning for Intensive Care Unit Length-of-Stay Prediction: A Simulation-Based Approach to Bed Capacity Management
Source: Med Decis Making. 2025 Dec 26;46(3):355–70. doi: 10.1177/0272989X251406639 (PMC12976099; doi:10.1177/0272989X251406639)
Supplement: sj-docx-2-mdm-10.1177_0272989X251406639 – Supplemental material for Machine Learning for Intensive Care Unit Length-of-Stay Prediction: A Simulation-Based Approach to Bed Capacity Management [file sj-docx-2-mdm-10.1177_0272989X251406639.docx]

**Appendix**

**Variable importance plots**


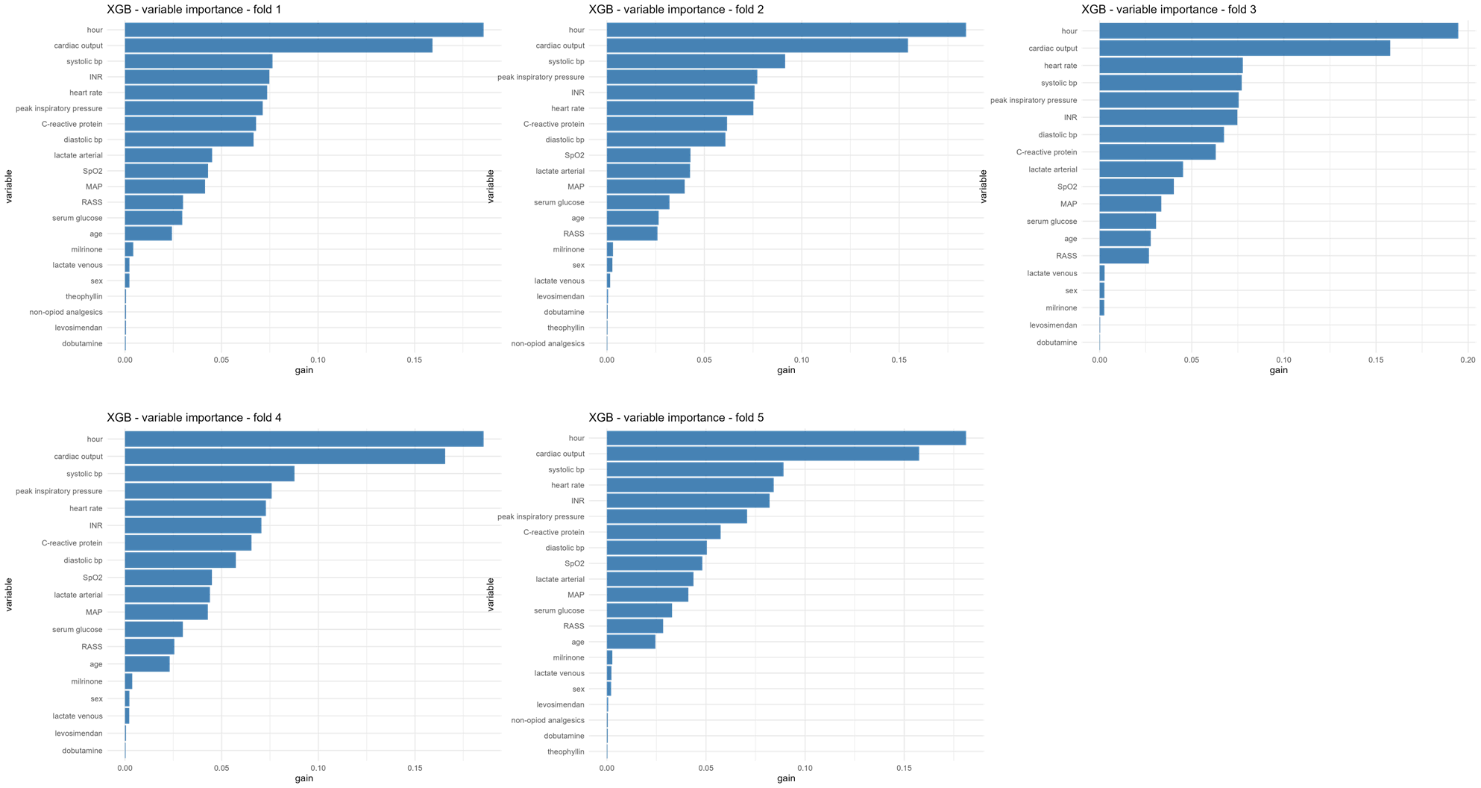


Figure 7: Variable importance plots for XGB models in $\tau=0$.


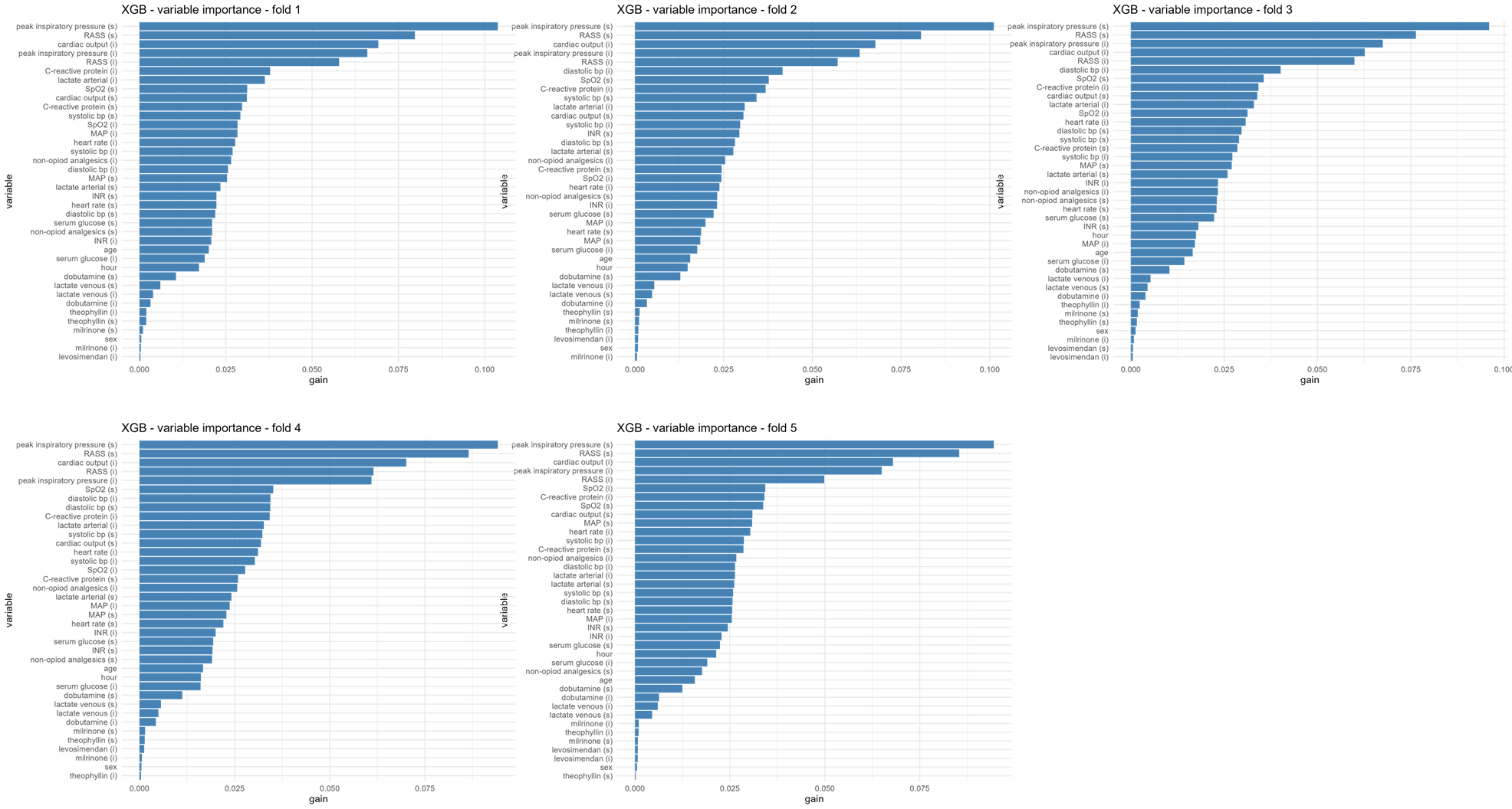


Figure 8: Variable importance plots for XGB models in $\tau=1$ (i – intercept, s – slope).


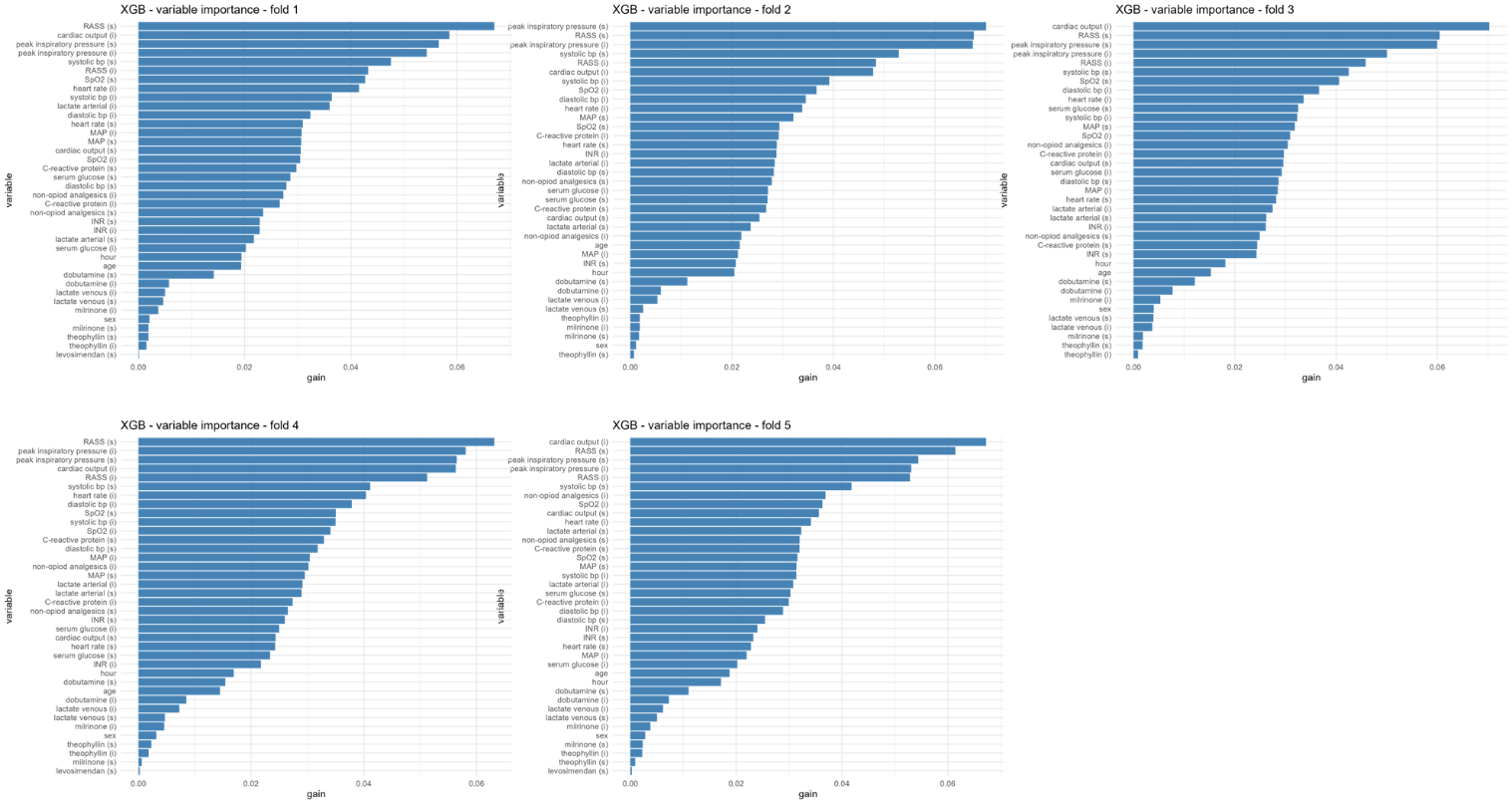


Figure 9: Variable importance plots for XGB models in $\tau=2$ (i – intercept, s – slope).


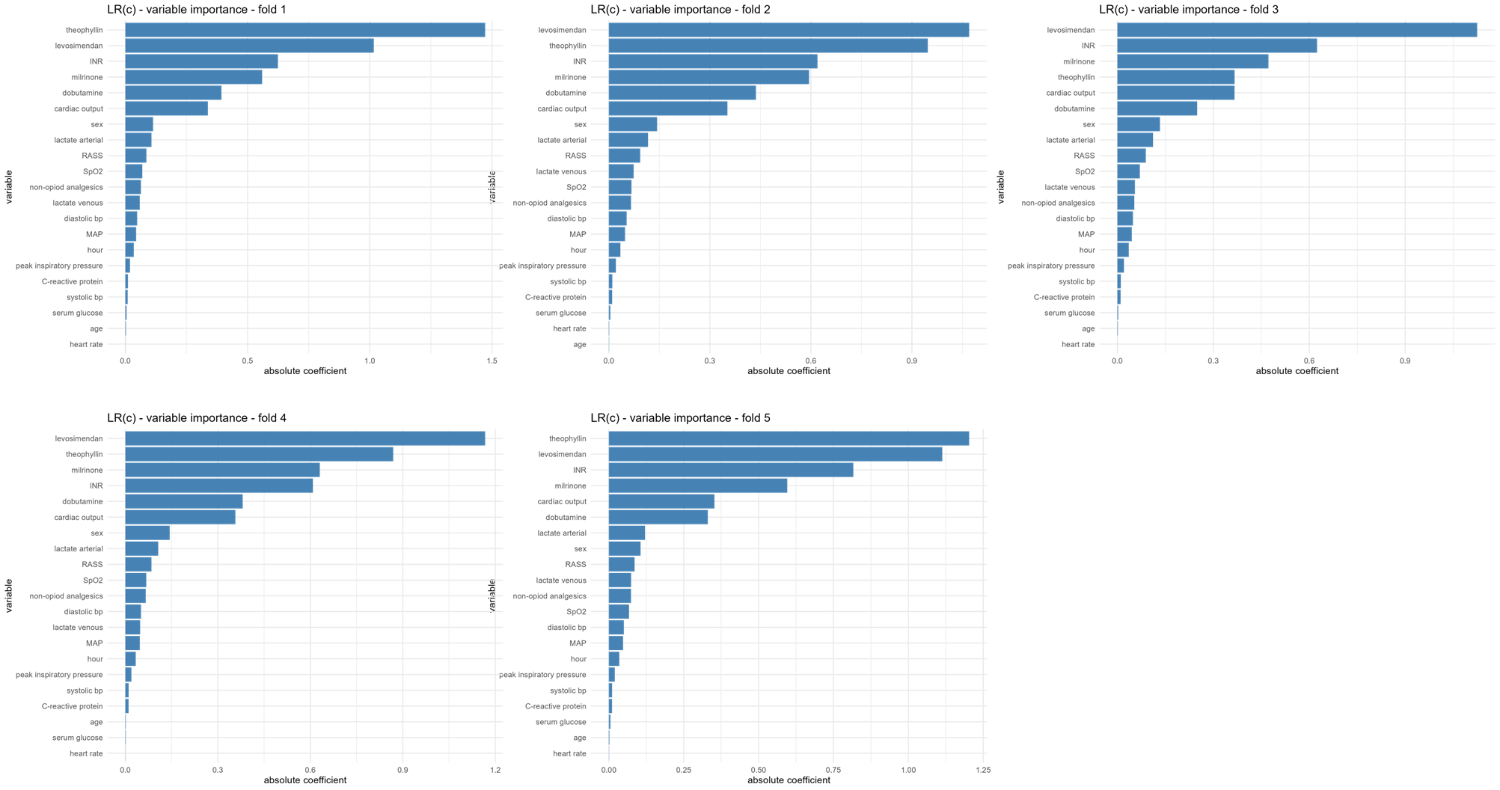


Figure 10: Variable importance plots for LR models (c – complex) in $\tau=0$.


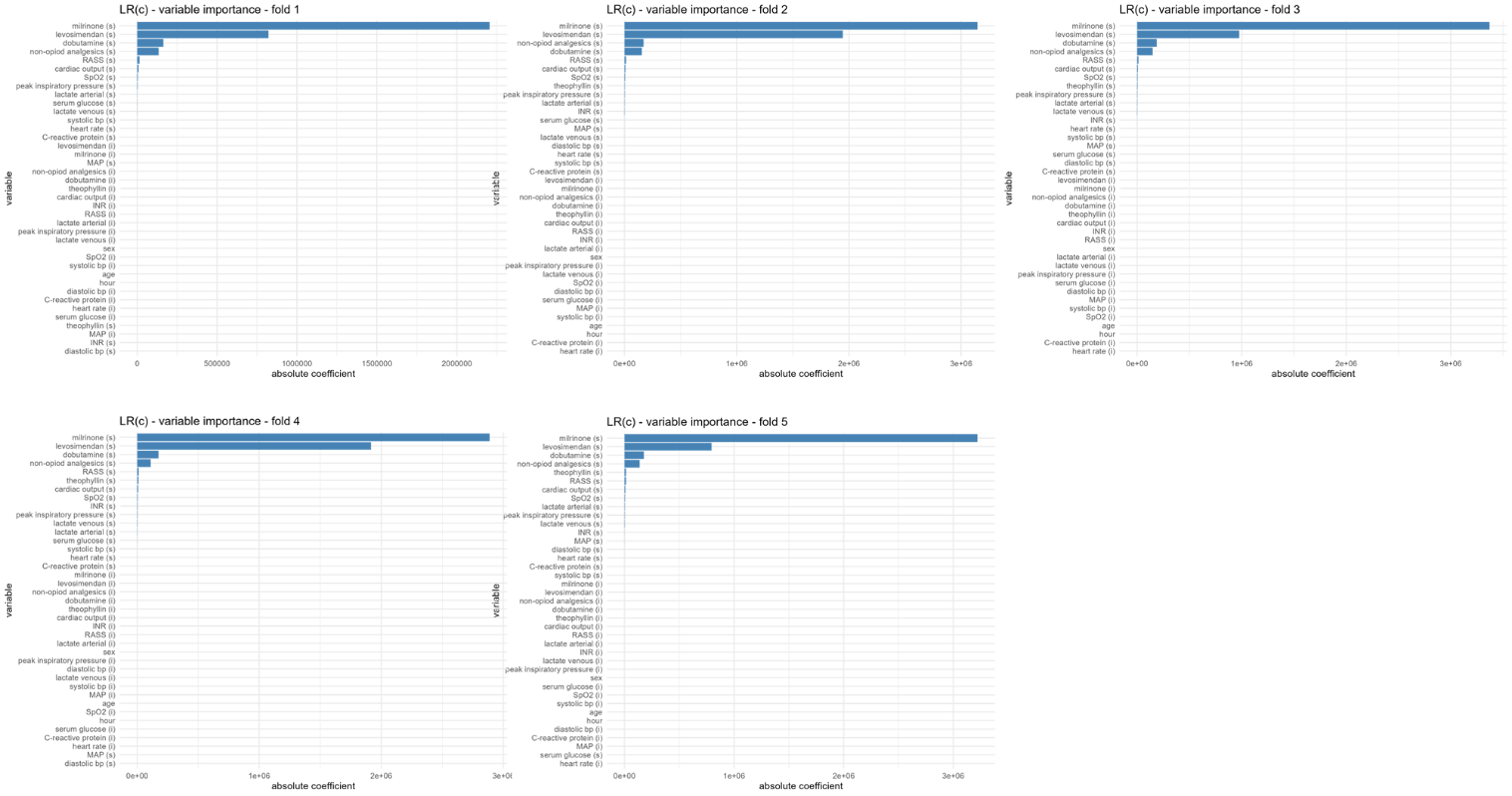


Figure 11: Variable importance plots for LR models (c – complex) in $\tau=1$ (i – intercept, s – slope).


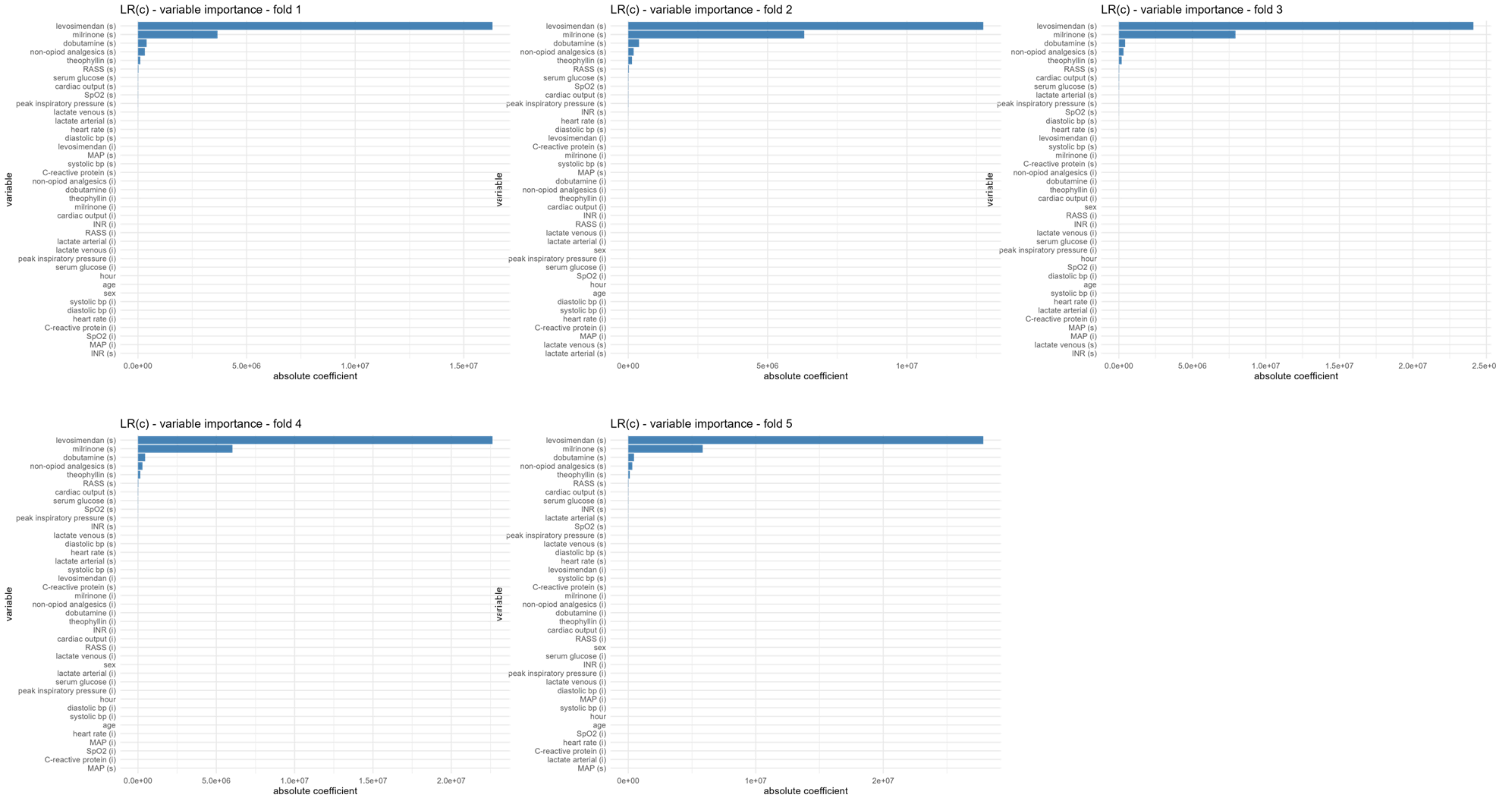


Figure 12: Variable importance plots for LR models (c – complex) in $\tau=2$ (i – intercept, s – slope).


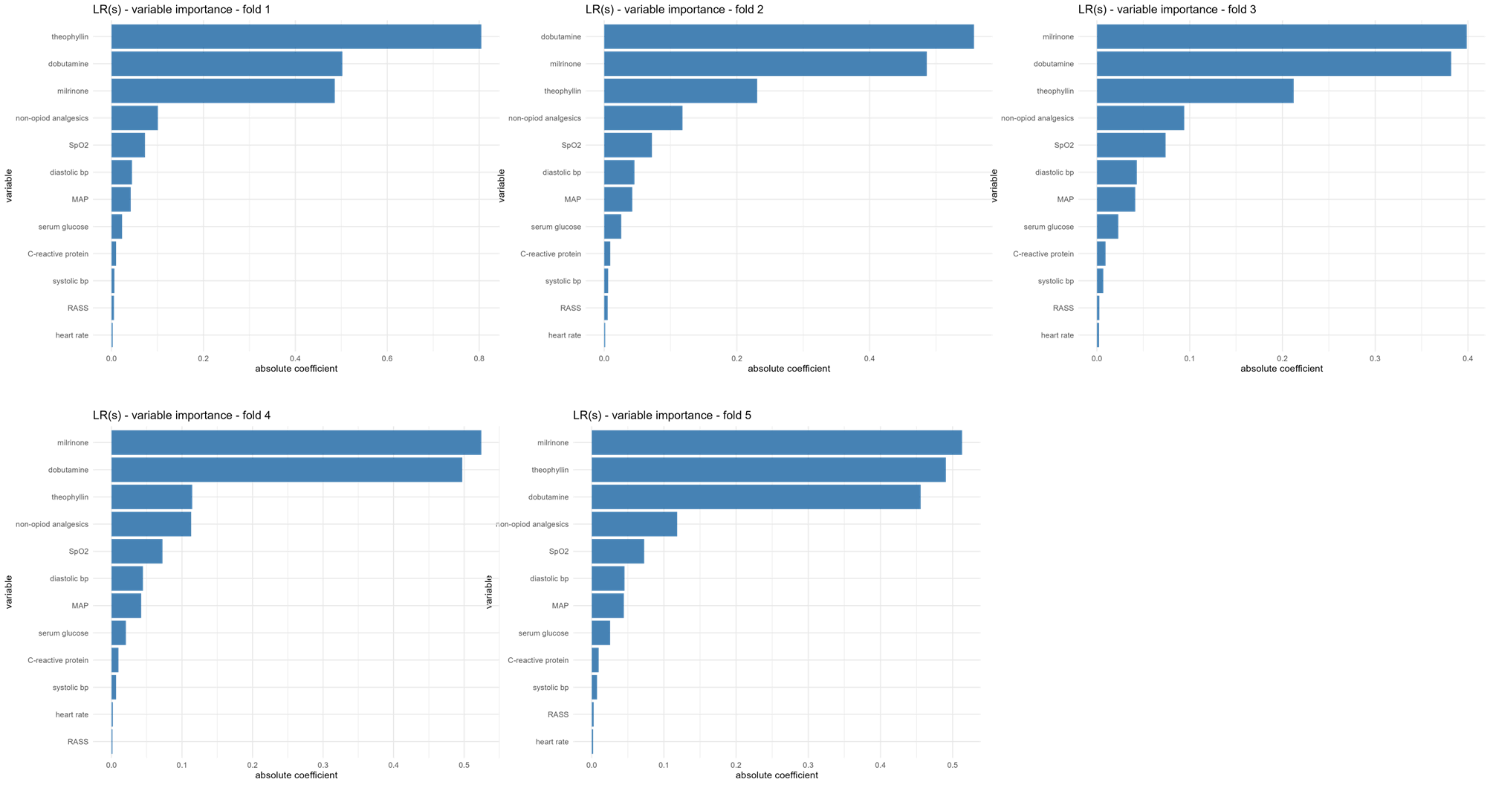


Figure 13: Variable importance plots for LR models (s – simple) in $\tau=0$.


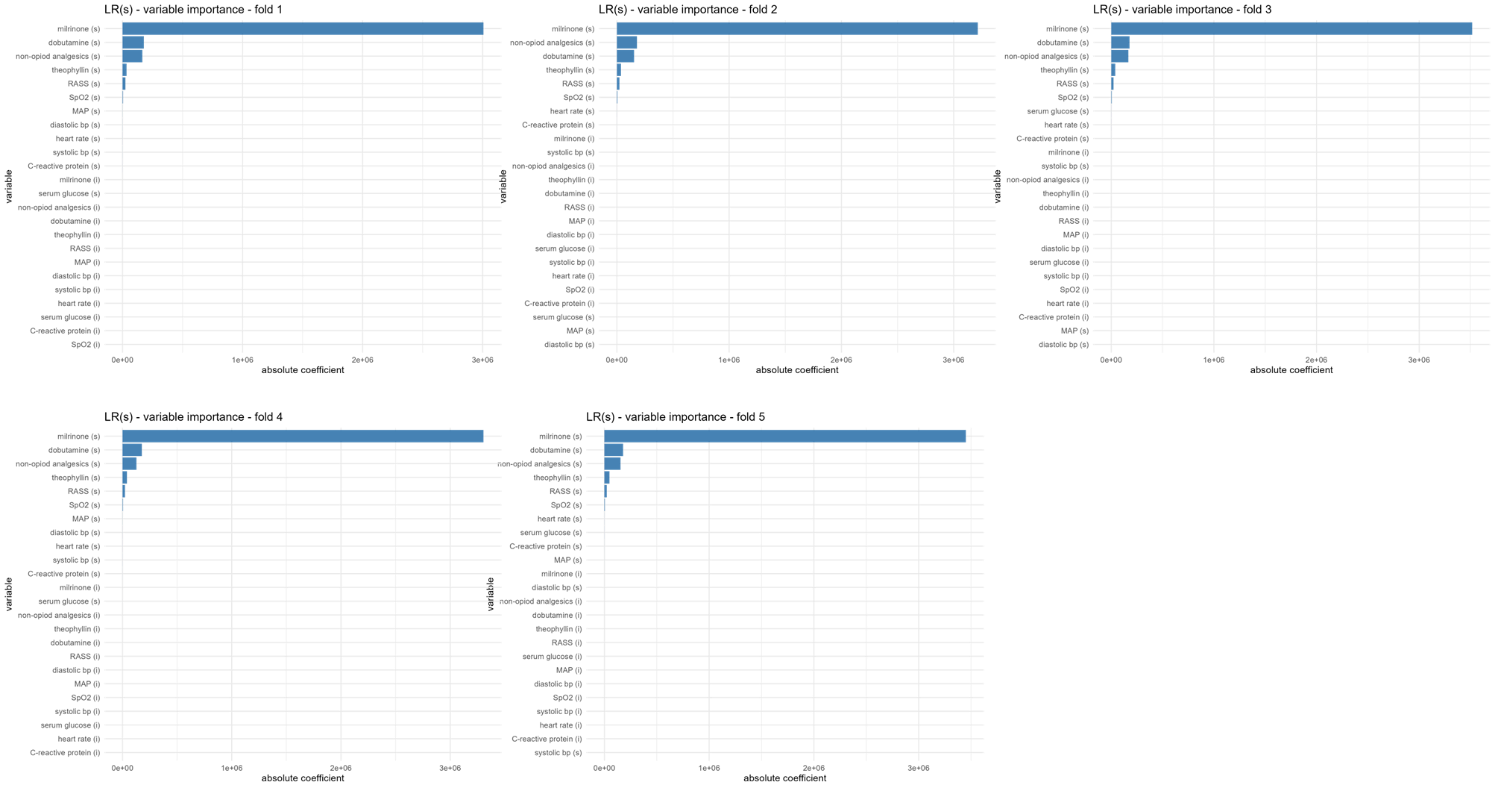


Figure 14: Variable importance plots for LR models (s – simple) in $\tau=1$ (i – intercept, s – slope).


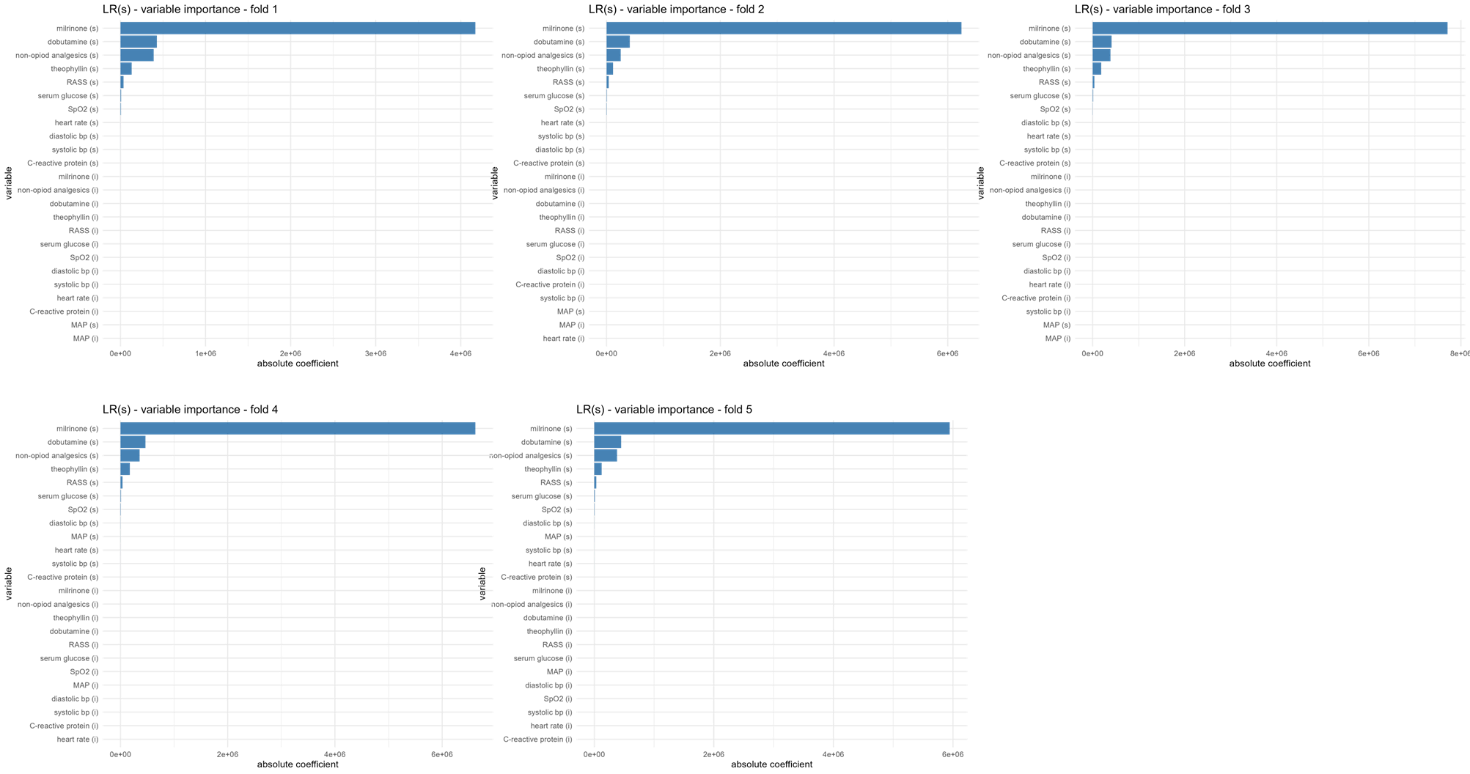


Figure 15: Variable importance plots for LR models (s – simple) in $\tau=2$ (i – intercept, s – slope).

- 1. **Calibration plots**


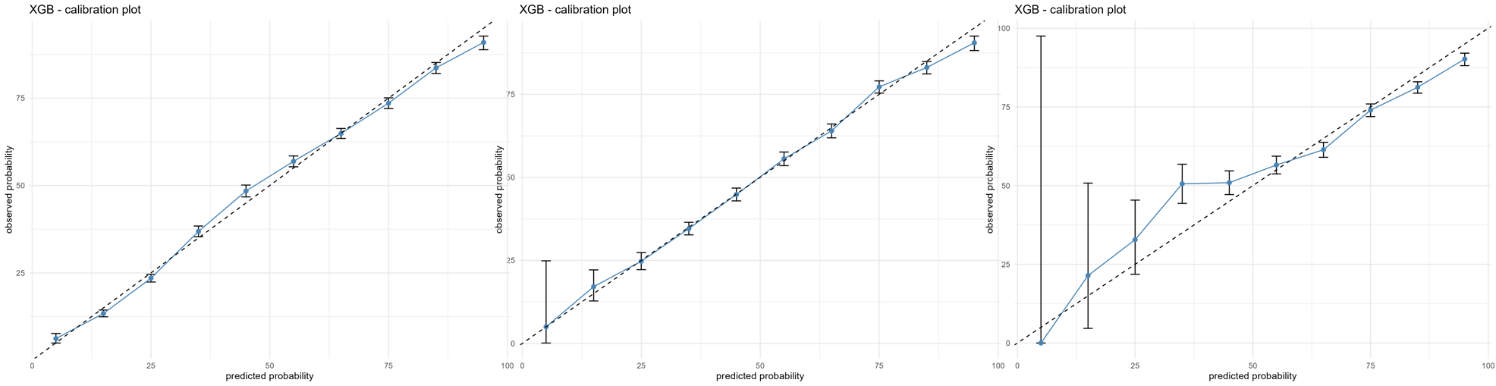


Figure 16: Calibration plots for XGB model in all timesteps


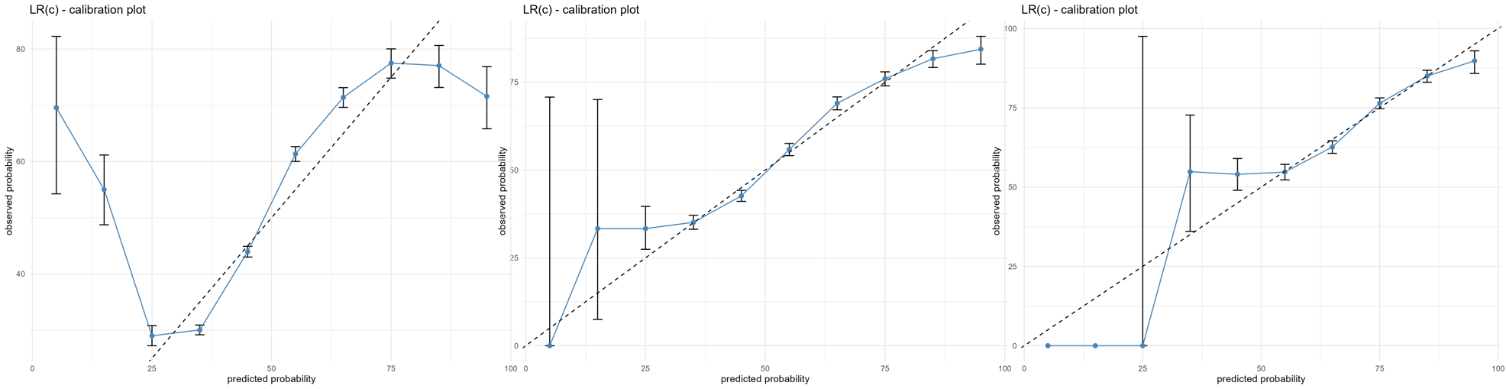


Figure 17: Calibration plots for LR models (c – complex) in all timesteps


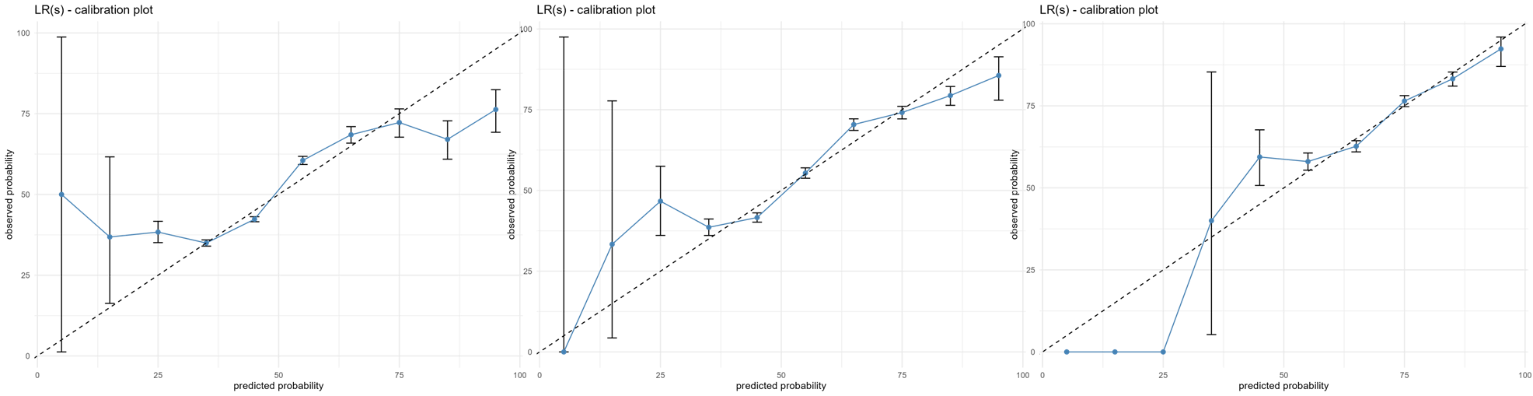


Figure 18: Calibration plots for LR models (s – simple) in all timesteps
